# Supplementary material for: Reactivation of Myc transcription in the mouse heart unlocks its proliferative capacity
Source: Nat Commun. 2020 Apr 14;11:1827. doi: 10.1038/s41467-020-15552-x (PMC7156407; doi:10.1038/s41467-020-15552-x)
Supplement: Supplementary file 1 — Supplementary Information [file 41467_2020_15552_MOESM1_ESM.pdf]

Supplementary Information

Reactivation of Myc transcription in the mouse heart unlocks its proliferative capacity

Bywater et al.

Correspondence to: chw39@cam.ac.uk and gie20@cam.ac.uk

This PDF file includes:

Supplementary Figures 1-9
Supplementary Table 1

Supplementary Figures:

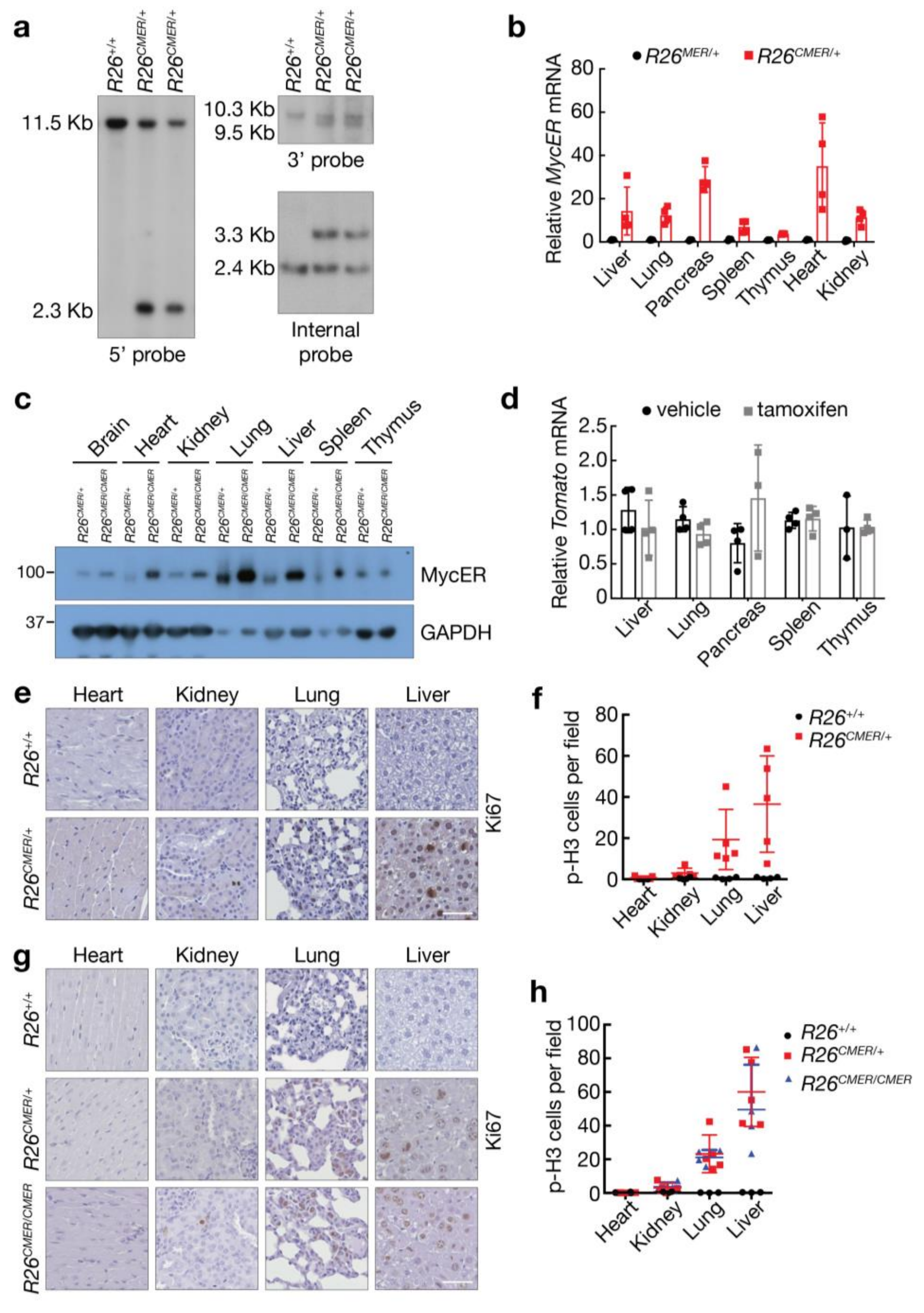

Supplementary Figure 1: Design and validation of a MycER^{TM} mouse

a. Southern blot analysis from *R26*$^{+/+}$ wild-type and *R26*$^{CMER/+}$ mice, analysed with 5′ (EcoRI), 3' (EcoRI/SalI) and internal (BamHI) probes. Wild-type fragments (11.5 kb, 5'; 10.3 kb, 3' and 2.4 kb, internal) targeted fragments (2.3 kb, 5'; 9.5 kb, 3' and 3.3 kb, internal). Representative results from 96 independent ES cell clones, later confirmed germline transmission in mice. **b.**

Quantitative RT-PCR analysis of *MycER* in *R26*$^{MER/+}$ (*n*=4) and *R26*$^{CMER/+}$ (*n*=4) liver, lung, pancreas, spleen, thymus, heart and kidney. Expression is relative to the respective *R26*$^{MER/+}$. Mean and s.e.m. shown. **c.** Immunoblot analysis of MycER^{T2} expression in the brain, heart, kidney, lung, pancreas, liver, spleen and thymus isolated from *R26*$^{CMER/+}$ and *R26*$^{CMER/CMER}$ mice. Represents the results from 2 individual mice. **d.** Quantitative RT-PCR analysis of *Tomato* in *R26*$^{CMER/mTmG}$ liver, lung, spleen, pancreas, and thymus (n=4, except pancreas tamoxifen and spleen oil n=3) isolated 4 hours post administration of oil or tamoxifen. Expression is relative to the respective vehicle control. Mean and s.e.m shown. **e.** Immunohistochemistry of Ki67 in the heart, kidney, lung and liver isolated from wild-type (*R26*$^{+/+}$) and *R26*$^{CMER/+}$ mice 72 hours post administration of tamoxifen. Representative images based on analysis of 4 independent mice. **f.** Quantification of p-H3 positive nuclei per field of view of the heart, kidney, lung and liver (positive hepatocyte nuclei only) isolated from wild-type (*R26*$^{+/+}$, *n*=4) and *R26*$^{CMER/+}$ (*n*=5) mice 72 hours post administration of tamoxifen. Means are taken from 5 images per mouse; Mean and s.d. shown. **g.** Immunohistochemical staining of Ki67 in the heart, kidney, lung and liver isolated from wild-type (*R26*$^{+/+}$), *R26*$^{CMER/+}$ and *R26*$^{CMER/CMER}$ mice 24 hours post administration of tamoxifen Representative images based on analysis of at least 3 independent mice. **h.** Quantification of p-H3 positive nuclei per field of view of the heart, kidney, lung and liver (positive hepatocyte nuclei only) isolated from wild-type (*R26*$^{+/+}$, *n*=3), *R26*$^{CMER/+}$ (*n*=5) and *R26*$^{CMER/CMER}$ (*n*=4) mice 24 hours post administration of tamoxifen. Means are taken from 5 images per mouse; Mean and s.d. shown. In all cases scale bars represents 50μm and replicate samples are derived from independent mice. Source data are provided as a Source Data file.

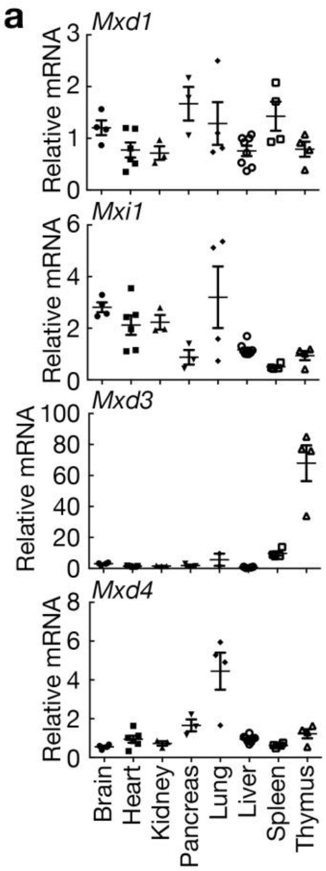

Supplementary Figure 2: Mxd expression across tissues

a. Quantitative RT-PCR analysis of *Mxd1, Mxi1, Mxd3 and Mxd4* in wild-type brain (n=4), heart (n=6), kidney (n=3), pancreas (n=4 except n=2 for *Mxd3*), lung (n=3), liver (n=8 except n=4 for *Mxd3*), spleen (n=4) and thymus (n=4). Expression is relative to liver. Mean and s.e.m. shown. Replicate samples are derived from independent mice. Source data are provided as a Source Data file.

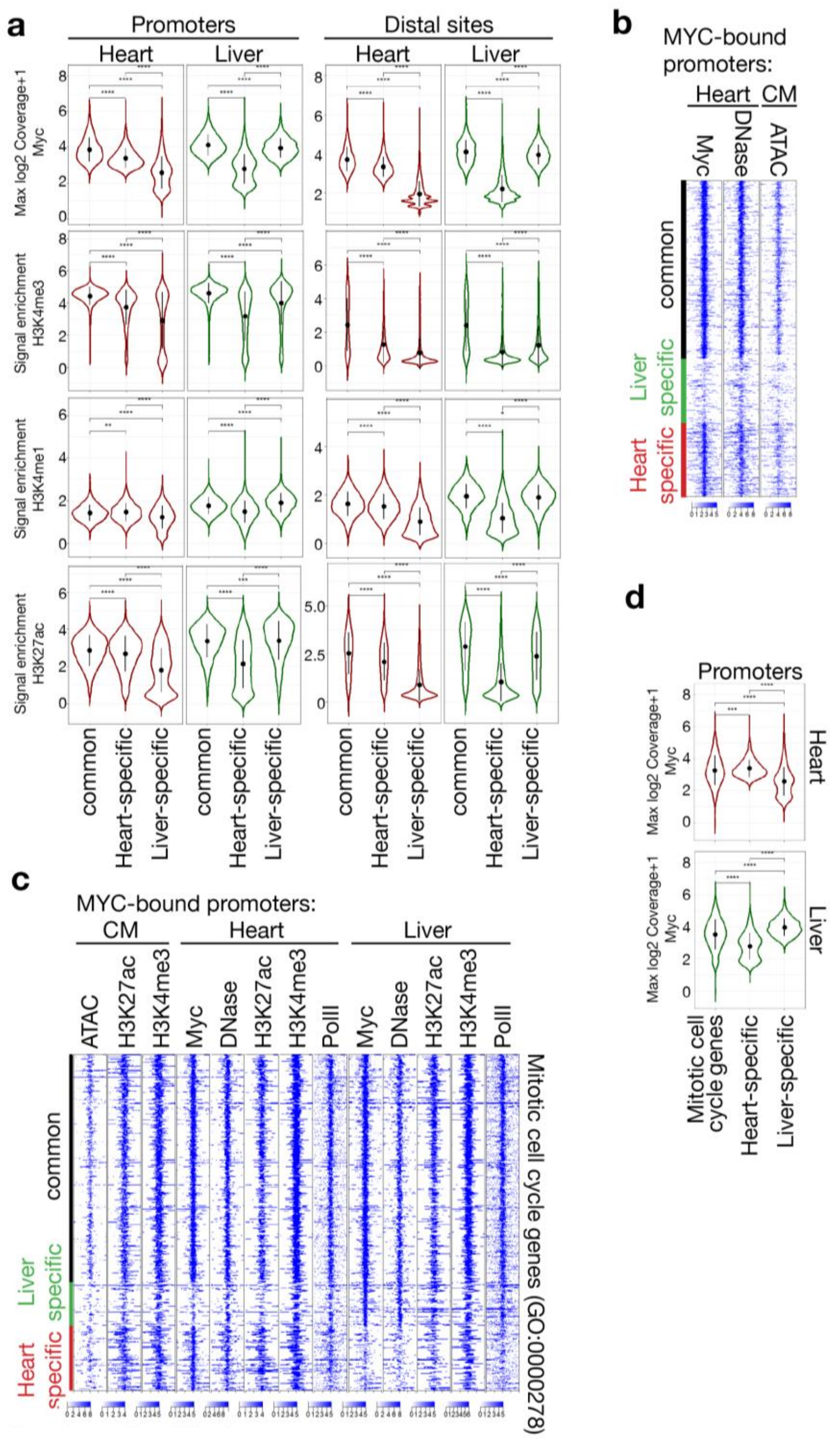

Supplementary Figure 3: Myc binding in the heart and liver

a. Violin plots providing quantitative visualization of the read coverage of Myc, H3K4me3, H3K27ac and H3K4me1 at promoter and distal elements of common versus heart or liver tissue specific sites. P-values are derived by pairwise two-sided Mann-Whitney P≤ 0.05*, P≤0.01**,

P≤0.001*** and P≤0.0001****. Plot shows distribution (violin), mean (black dot) and standard deviation (bar). **b.** Heat map of peaks called for ATAC seq of cardiomyocytes, DNAse treated chromatin from whole heart and Myc ChIP sequencing on whole heart at Myc-bound promoter elements that are common between both the liver and heart (common-black) or specific for an individual tissue (liver specific-green, heart specific-red). Myc ChIP sequencing performed on the heart isolated from *R26*$^{CMER/+}$ mice 4 hours post administration of 4-OHT, overlap of n=2. **c.** Heat map of peaks called for ATAC seq, acetylated H3K27 (H3K27ac) and tri-methylated H3K4 (H3K4me3) ChIP sequencing on purified cardiomyocytes (CM) compared to peaks called by DNAse treated, acetylated H3K27 (H3K27ac), tri-methylated H3K4 (H3K4me3) and RNA Polymerase II (PolII) ChIP sequencing on whole heart (Heart) and liver (Liver) at Mitotic Cell Cycle genes (GO Biological process gene set 0000278) with Myc-bound promoter elements that are common between both the liver and heart (common-black) or specific for an individual tissue (liver specific-green, heart specific-red). Myc ChIP sequencing was performed on the heart and livers isolated from *R26*$^{CMER/+}$ mice 4 hours post administration of 4-OHT, overlap of n=2. PolII ChIP sequencing performed on the heart and livers isolated from wild-type (*R26*$^{+/+}$) mice, overlap of n=2. **d.** Violin plots providing quantitative visualization of the read coverage of Myc at promoters of Mitotic cell cycle genes (GO: 0000278) and promoters specifically bound by Myc in the heart or liver. P-values are derived by pairwise two-sided Mann-Whitney P≤0.001*** and P≤0.0001****. Plot shows distribution (violin), mean (black dot) and standard deviation (bar). ChIP sequencing data for DNase treated, H3K27ac and H3K4me3 were taken from the ENCODE Project (accession numbers; GSM1014166, GSM1000093, GSM769017, GSM1014195, GSM1000140, GSM769014), cardiomyocyte ATAC sequencing accession: GSE95763, cardiomyocyte H3K27ac and H3K4me3 ChIP sequencing accession: SRP033385. Source data are provided as a Source Data file.

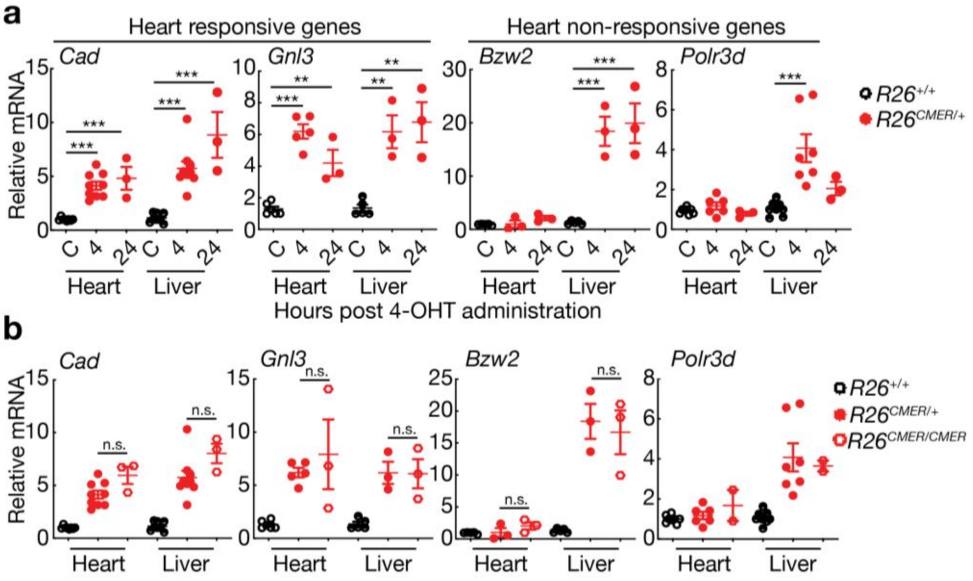

Supplementary Figure 4: The transcriptional responses to activation of ectopic Myc

a. Quantitative RT-PCR analysis of *Cad, Gnl3, Bzw2 and Polr3d* in hearts and livers isolated from wild-type ($R26^{+/+}$, *n*=8 *Cad, Polr3d*, n=5 *Gnl3, Bzw2*) and $R26^{CMER/+}$ (*n*=9 *Cad*, n=5 heart *Gnl3*, n=5 liver *Gnl3*, n=3 *Bzw2*, n=6 heart *Polr3d*, n=7 liver *Polr3d*) mice at either 4 or 24 (n=3) hours post administration of 4-OHT. Expression is relative to the respective control (C). Mean and s.e.m. shown. One Way ANOVA with Tukey's multiple comparisons test; heart $R26^{+/+}$ control vs 4 hour $R26^{CMER/+}$: P<0.001*** (*Gnl3*), P<0.01** (*Cad*), heart $R26^{+/+}$ control vs 24 hour $R26^{CMER/+}$: P<0.05* (*Gnl3*), P<0.01** (*Cad*), liver $R26^{+/+}$ control vs 4 hour $R26^{CMER/+}$: P<0.001*** (*Gnl3*, *Cad, Bzw2, Polr3d*), liver $R26^{+/+}$ control vs 24 hour $R26^{CMER/+}$: P<0.001*** (*Gnl3, Cad*, *Bzw2*). Replicate samples are derived from independent mice. **b.** Quantitative RT-PCR analysis of *Cad, Gnl3, Bzw2 and Polr3d* in hearts isolated from wild-type ($R26^{+/+}$, n as above), $R26^{CMER/+}$ (n as above) and $R26^{CMER/CMER}$ (n=3 except *Polr3d* n=2) mice 4 hours post administration of 4-OHT. Expression relative to the wild-type control. Mean and s.e.m. shown. One-Way ANOVA with Tukey's multiple comparisons test; P>0.05$^{n.s.}$ for all comparisons between $R26^{CMER/+}$ and $R26^{CMER/CMER}$. Replicate samples are derived from independent mice. Source data are provided as a Source Data file.

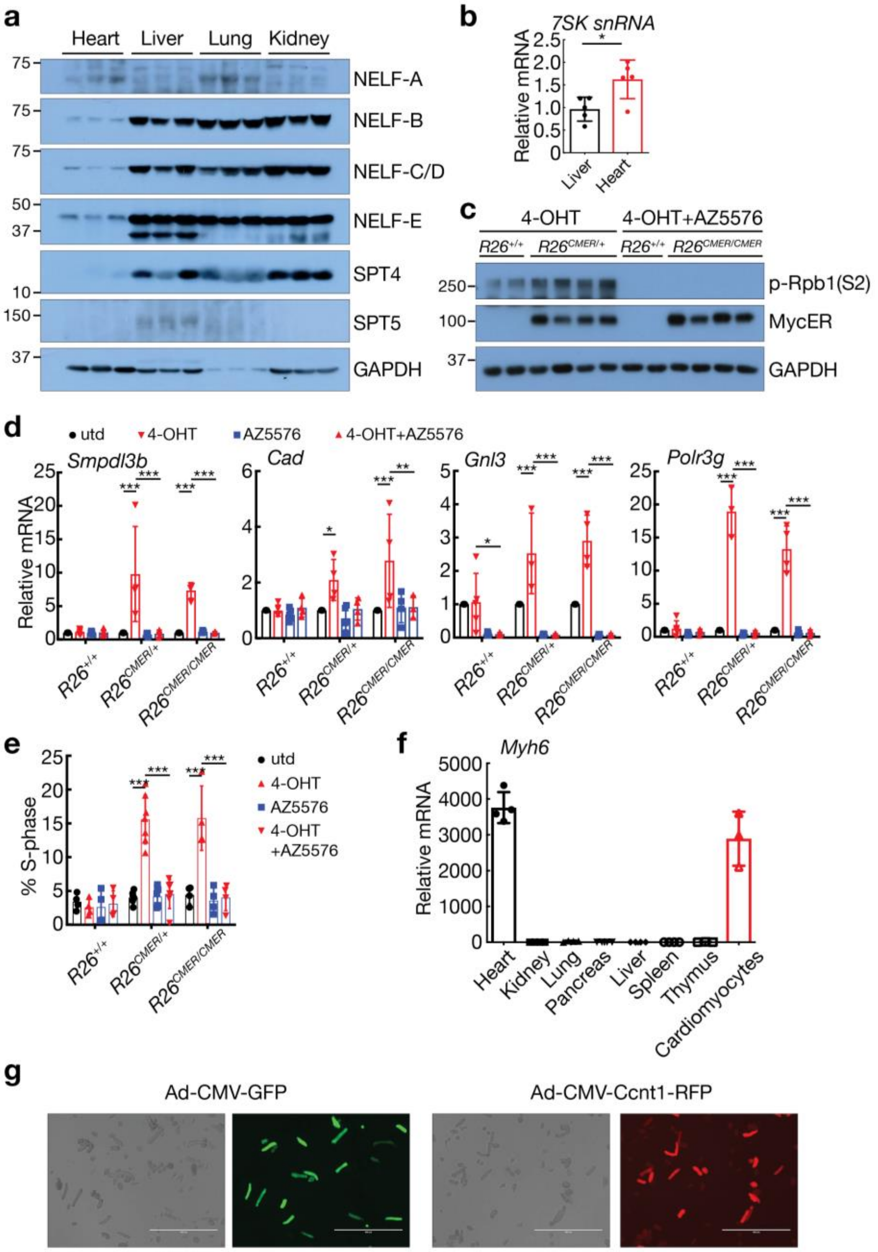

Supplementary Figure 5: The transcriptional activity of Myc is limited by P-TEFb availability

a. Immunoblot analysis of NELF-A, NELF-B, NELF-C/D, NELF-E, SPT4 and SPT5 expression in the heart, liver, lung and kidney isolated from wild-type (*R26*$^{+/+}$) mice. **b.** Quantitative RT-PCR analysis of *7SK snRNA* in liver and heart isolated from wild-type mice (*n*=5). Expression is relative to liver. Mean and s.e.m. shown. Unpaired t-test; liver vs heart P= 0.019*. **c.** Immunoblot analysis of the C-terminal domain of RNA Polymerase II, phosphorylated (p-Rpb1(S2)), and MycER^{T2} protein expression in wild-type (*R26*$^{+/+}$), *R26*$^{CMER/+}$ or *R26*$^{CMER/CMER}$

MEFs in culture, isolated four hours post addition of 100 nM 4-OHT either alone (4-OHT) or in combination with 1μM AZ-5576 (4-OHT+AZ5576).. **d.** Quantitative RT-PCR analysis of *Smpdl3b, Cad, Gnl3* and *Polr3g* in wild-type ($R26^{+/+}$, *n*=5, except 4-OHT+AZ5576 *n=4*), $R26^{CMER/+}$ (*Smpdl3b, Cad; n*=5, except 4-OHT+AZ5576 and 4OHT *n=4. Gnl3* and *Polr3g n=4* except 4-OHT *n=3*) or $R26^{CMER/CMER}$ (*n*=4, except 4-OHT+AZ5576 *n=3*) MEFs cultured in serum deplete media, either untreated (utd) or isolated 4 hours post addition of 100 nM 4-OHT or 1 μM AZ-5576 (AZ5576) either alone or in combination (4-OHT+AZ5576). Expression is relative to the respective untreated control (utd). Mean and s.d. shown. Two Way ANOVA with Tukey's multiple comparisons test; utd vs 4-OHT: *P*=0.001*** (*Smpdl3b*: $R26^{CMER/+}$, $R26^{CMER/CMER}$; *Cad*: $R26^{CMER/CMER}$*; Gnl3:* $R26^{CMER/CMER}$*; Polr3g:* $R26^{CMER/+}$, $R26^{CMER/CMER}$), 0.05* (*Cad*: $R26^{CMER/+}$*; Gln3:* $R26^{CMER/+}$); 4-OHT vs 4-OHT+AZ5576: *P*=0.001*** (*Smpdl3b*: $R26^{CMER/+}$, $R26^{CMER/CMER}$*; Gnl3:* $R26^{CMER/+}$, $R26^{CMER/CMER}$*; Polr3g:* $R26^{CMER/+}$, $R26^{CMER/CMER}$), 0.01** (*Cad*: $R26^{CMER/CMER}$).. **e.** Flow cytometry analysis of DNA content in wild-type ($R26^{+/+}$, *n*=4), $R26^{CMER/+}$ (*n*=7) or $R26^{CMER/CMER}$ (*n*=4) MEFs cultured in serum deplete media, either untreated (utd) or isolated 16 hours post addition of 100 nM 4-OHT or 1μM AZ-5576 (AZ5576) either alone or in combination (4-OHT+AZ5576). Mean and s.d. shown Two Way ANOVA with Tukey's multiple comparisons test; utd vs 4-OHT: *P*=0.001*** ($R26^{CMER/+}$, $R26^{CMER/CMER}$); 4-OHT vs 4-OHT+AZ5576: *P*=0.001*** ($R26^{CMER/+}$, $R26^{CMER/CMER}$). **f.** Quantitative RT-PCR analysis of *Myh6* in the heart (*n*=4), kidney (*n*=4), lung (*n*=4), pancreas (*n*=4), liver (*n*=4), spleen (*n*=4) and thymus (*n*=3) of wild-type mice, and cardiomyoctyes (*n*=3) isolated from wild-type mice. Expression is relative to liver. Mean and s.e.m. shown. Replicate samples are derived from independent mice. **g.** Phase contrast and fluorescent images of adult cardiomyocytes in culture 48 hours post infection. GFP (Ad-CMV-GFP) or RFP (Ad-AMV-Ccnt1-RFP). Scale bar is 400 μm. Representative of infections of 10 independent cardiomyocyte isolations. In all cases, replicate samples are derived from independent mice or MEF lines. Source data are provided as a Source Data file.

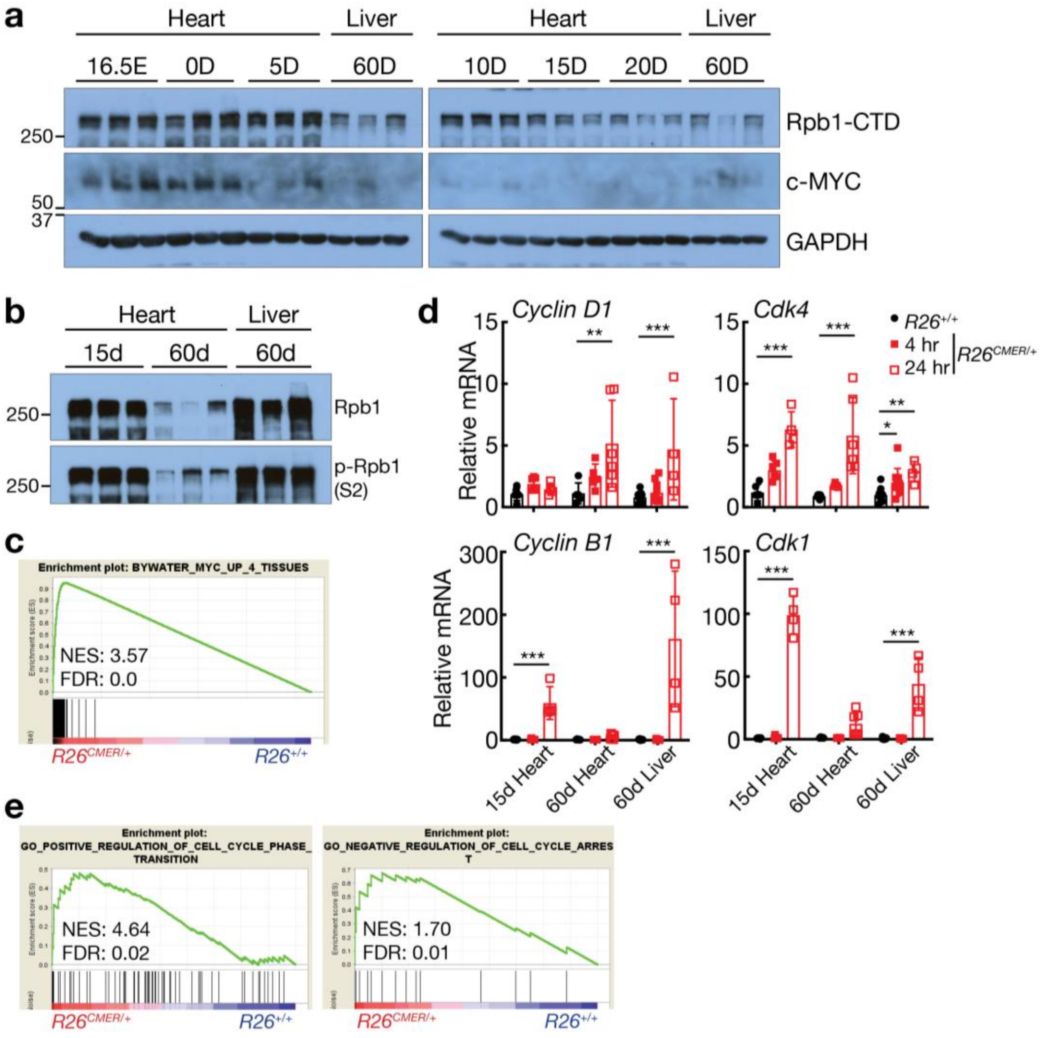

Supplementary Figure 6: The juvenile heart is permissive for Myc-driven transcriptional activation

a. Immunoblot analysis of the C-terminal domain of RNA Polymerase II, total (Rpb1) and c-Myc protein expression in wild-type heart isolated from embryos at 16.5 days post fertilisation (E16.5), neonates at birth (0D), 5 day (5D), 10 day (10D) 15 day (15D) and 20 day (20D) old mice and wild-type liver isolated from 60 day (60D) old mice. Replicate samples are derived from independent mice. **b.** Longer exposure of Immunoblot shown in Figure. 5a, showing the C-terminal domain of RNA Polymerase II and total (Rpb1) and phosphorylated (p-Rpb1(S2)) expression in wild-type heart and liver isolated from 15 day-old (15d) and 60 day-old (60d) mice. **c.** Enrichment of common Myc targets (liver, lung, kidney and heart) in heart isolated from 15

day *R26*$^{CMER/+}$ mice in comparison to controls (*R26*$^{+/+}$) at 4 hours post administration of 4-OHT, as determined by RNA sequencing. Including normalised enrichment score (NES) and FDR q-value (FDR). **d.** Quantitative RT-PCR analysis of *Cyclin D1, Cdk4, Cyclin B1 and Cdk1* in wild-type (*R26*$^{+/+}$, *n*=6 15d heart, n=5 60d heart (except *Cdk4* n=7), n=13 60d liver) and *R26*$^{CMER/+}$ heart isolated from 15 day-old (15d Heart) mice versus heart and liver isolated from 60 day-old (60d Heart)(60d Liver) mice 4 (light grey, n=6 15d heart, n=5 60d heart, n=10 60d liver) or 24 (dark grey, n=4 15d heart, n=6 60d heart, n=4 60d liver) hours post i.p. of 4-OHT. Expression is relative to the respective wildtype (*R26*$^{+/+}$). Mean and s.d. shown. One Way ANOVA with Tukey's multiple comparisons test; 15d heart *R26*$^{+/+}$ vs 24 hour *R26*$^{CMER/+}$: P=0.001*** (*Cdk4, Cyclin B1* and *Cdk1*), 60d heart *R26*$^{+/+}$ vs 24 hour *R26*$^{CMER/+}$: P=0.01** (*Cyclin D1*) P=0.001*** (*Cdk4*), 60d liver *R26*$^{+/+}$ vs 4 hour *R26*$^{CMER/+}$: P=0.05* (*Cdk4*), 60d liver *R26*$^{+/+}$ vs 24 hour *R26*$^{CMER/+}$: P=0.01** (*Cdk4*) P=0.001*** (*Cyclin D1, Cyclin B1, Cdk1*). Replicate samples are derived from independent mice. **e.** Enrichment of pro-cell cycle progression gene sets in heart isolated from 15 day *R26*$^{CMER/+}$ mice in comparison to controls (*R26*$^{+/+}$) at 4 hours post administration of 4-OHT, as determined by RNA sequencing. Including normalised enrichment score (NES) and FDR q-value (FDR). Source data are provided as a Source Data file.

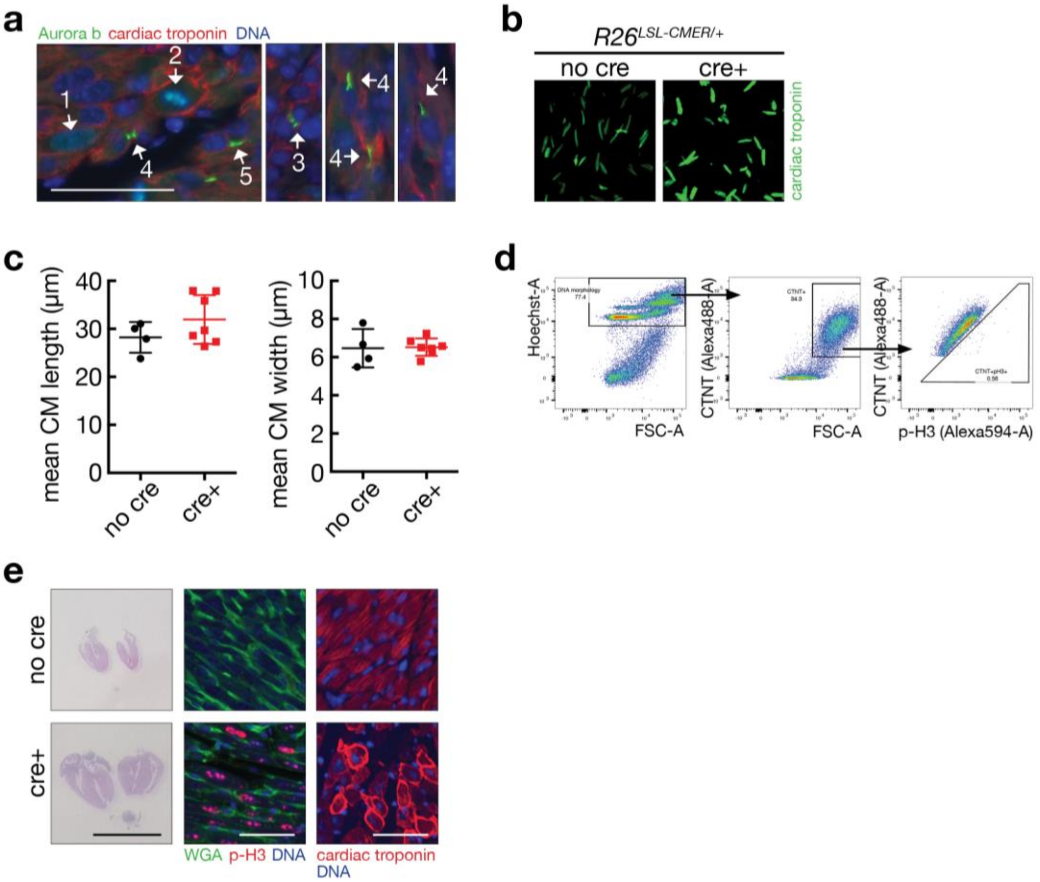

Supplementary Figure 7: The juvenile heart is permissive to Myc-driven proliferation

a. Immunofluorescent staining of cardiac troponin and Aurora B in the heart of 15 day-old *Myh6-Cre*; $R26^{LSL\text{-}CMER/+}$ mice 48 hours post administration of tamoxifen. Arrows show differences in Aurora B localization throughout the cell cycle. 1- G2, nucleus. 2- Metaphase, metaphase chromosomes. 3- Anaphase, mid-zone. 4- Cytokinesis, centrally located mid-body. 5- Failing cytokinesis- laterally displaced mid-body. Representative images from 3 mice. Scale bar represents 50μm. **b.** Immunofluorescent staining of cardiac troponin T (green) positive cardiomyocytes isolated from fixed and dissociated hearts of 15 day-old control ($R26^{LSL\text{-}CMER/+}$; no cre) and *Myh6-Cre*; $R26^{LSL\text{-}CMER/+}$ ($R26^{LSL\text{-}CMER/+}$;cre+) mice. Representative of staining conducted on cardiomyocytes purified 11 independent mice. **c.** Quantification of the length and width of cardiomyocytes isolated from the hearts of 15 day-old control ($R26^{LSL\text{-}CMER/+}$; no cre, n=4) and *Myh6-Cre*; $R26^{LSL\text{-}CMER/+}$ ($R26^{LSL\text{-}CMER/+}$;cre+, n=6) mice 48 hours post administration of

tamoxifen. Mean determined from the measurement of >13 individual cardiomyocytes per mouse. Mean and s.e.m. shown. One-way Mann Whitney test; no cre vs cre+ P= not significant. Replicate samples are derived from independent mice. **d.** The gating strategy used to determine p-H3 positivity in cardiac troponin (CTNT) positive cardiomyocytes shown in Figure 5h. **e.** Top-H&E staining, middle-WGA and p-H3 immunofluorescent staining, bottom- cardiac troponin immunofluorescent staining of hearts from 15 day-old control ($R26^{LSL\text{-}CMER/+}$; no cre) and *Myh6-Cre*; $R26^{LSL\text{-}CMER/+}$ ($R26^{LSL\text{-}CMER/+}$;cre+) mice 96 hours post administration of tamoxifen. Representative of staining conducted on sections from 8 independent mice. Scale bars represents 1cm (whole heart) and 50μm. Source data are provided as a Source Data file.

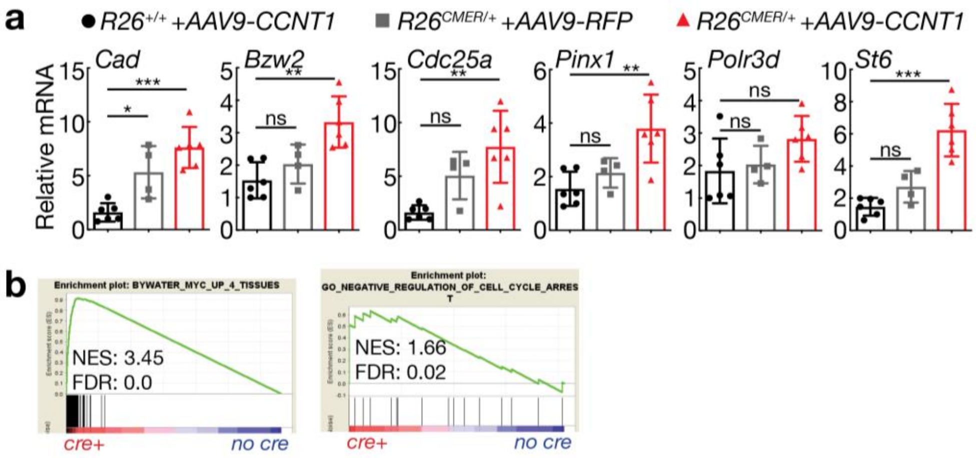

Supplementary Figure 8: Ccnt1 overexpression enables Myc driven transcription in adult cardiomyocytes

a. Quantitative RT-PCR analysis of *Cad, Bzw2, Pinx1, Polr3d, St6* and *Cdc25a* expression in adult wildtype (*R26*$^{+/+,}$ n=6) and *R26*$^{CMER/+}$ mouse heart isolated 4 weeks post systemic infection with an adeno associated virus encoding either *RFP* (*AAV9-RFP,* n=4) or *Ccnt1* (*AAV9-Ccnt1,* n=6) and 4 hours post administration of 4-OHT. Expression is relative to the respective wild-type*.* Mean and s.e.m. shown. One-Way ANOVA with Tukey's multiple comparisons test; *R26*$^{+/+}$ *AAV9-Ccnt1* vs *R26*$^{CMER/+}$ *AAV9-RFP*: P=0.05* (*Cad*). *R26*$^{+/+}$ *AAV9-Ccnt1* vs *R26*$^{CMER/+}$ *AAV9-Ccnt1*: P=0.01** (*Bzw2, Cdc25a* and *Pinx1*). *R26*$^{+/+}$ *AAV9-Ccnt1* vs *R26*$^{CMER/+}$ *AAV9-Ccnt1*: P=0.001*** (*Cad* and *St6*).Replicate samples are derived from independent mice. **b.** Top- enrichment of common Myc targets (liver, lung, kidney and heart), bottom- enrichment of a pro-cell cycle progression gene set in heart isolated from adult *Myh6-Cre*; *R26*$^{LSL\text{-}CMER/+}$ (cre+) in comparison to control (*R26*$^{LSL\text{-}CMER/+}$, no cre), both treated with AAV-CCNT1 4 weeks before administration of 4-OHT (4hr), as determined by RNA sequencing. Including normalised enrichment score (NES) and FDR q-value (FDR). Source data are provided as a Source Data file.

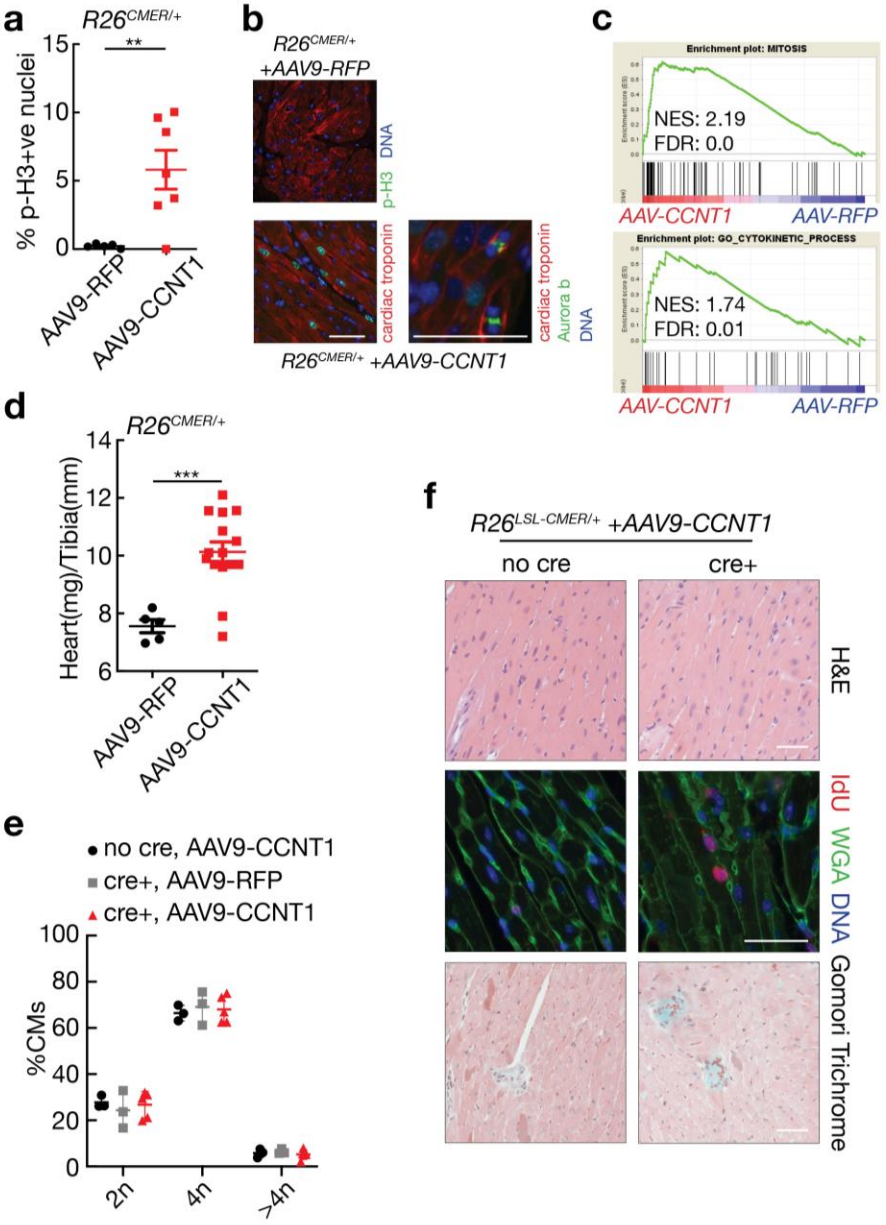

Supplementary Figure 9: Ccnt1 overexpression enables Myc-driven proliferation in adult cardiomyocytes

a. Quantification of p-H3 positive nuclei per field of view from adult *R26*$^{CMER/+}$ mouse hearts isolated 4 weeks post systemic infection with an adeno associated virus encoding either *RFP* (*AAV9-RFP,* n=5) or *Ccnt1* (*AAV9-Ccnt1,* n=7) and 48 hours post administration of tamoxifen. Means are taken from 5 images per mouse; Mean and s.e.m. shown. Unpaired t-test; *AAV9-RFP* vs *AAV9-Ccnt1,* P=0.0110. **b.** Immunofluorescent staining of cardiac troponin (red), p-H3 (green, left) and Aurora B positive mitotic nuclei (green, right) from adult *R26*$^{CMER/+}$ mouse

hearts isolated 4 weeks post infection with an adeno associated virus encoding either *RFP* (*AAV9-RFP*) or *Ccnt1* (*AAV9-Ccnt1*) and 48 hours post administration of tamoxifen. Representative images based on analysis of 5 independent mice. **c.** Enrichment of mitosis and cytokinesis gene sets in heart isolated from adult *Myh6-Cre*; $R26^{LSL\text{-}CMER/+}$ mice 4 weeks post systemic infection with AAV-CCNT1 verses AAV-*RFP* and 48 hours post administration of tamoxifen, as determined by RNA sequencing. Including normalised enrichment score (NES) and FDR q-value (FDR). **d.** The weight (mg) of hearts isolated from adult $R26^{CMER/+}$ mouse hearts isolated 4 weeks post infection with an adeno associated virus encoding either *RFP* (*AAV9-RFP,* n=5) or *Ccnt1* (*AAV9-Ccnt1,* n=15) and 48 hours post administration of tamoxifen, expressed as fold change over the length (mm) of a tibia isolated from the same mouse. Mean and s.e.m. shown. Unpaired t-test; *AAV9-RFP* vs *AAV9-Ccnt1,* P=0.0006. Replicate samples are derived from independent mice. **e.** Nuclei quantification performed on images of cardiomyocytes isolated from control ($R26^{LSL\text{-}CMER/+}$*;* no cre, n=3) and *Myh6-Cre*; $R26^{LSL\text{-}CMER/+}$ ($R26^{LSL\text{-}CMER/+}$*;*cre+) adult mouse hearts isolated 4 weeks post infection with an adeno associated virus encoding *Ccnt1* (*AAV9-Ccnt1*, n=5) or RFP (*AAV9-RFP*, n=3) and 72 hours post administration of tamoxifen. Mean and s.d. shown. One-Way Mann Whitney test; no cre vs cre+ P= 0.0043**. Replicate samples are derived from independent mice. **f.** Left- H&E staining, middle- IdU (BrdU) and WGA immunofluorescent staining and, right- Gomori Trichrome staining from control ($R26^{LSL\text{-}CMER/+}$*;* no cre) and *Myh6-Cre*; $R26^{LSL\text{-}CMER/+}$ ($R26^{LSL\text{-}CMER/+}$*;*cre+) adult mouse heart. Mice infected with an adeno associated virus encoding *Ccnt1* (*AAV9-Ccnt1*) at 4 weeks of age, given a single injection of tamoxifen to transiently activate MycER at 8 weeks of age and collected at 16 weeks of age. Representative of staining from 7 independent mice. Scale bars represent 50μm. Source data are provided as a Source Data file.

Supplementary Table 1. qPCR primers.

Oligonucleotides	SOURCE	IDENTIFIER
Ccnd1; forward 5'- GCGTACCCTGACACCAATCTC -3', reverse 5'- CTCCTCTTCGCACTTCTGCTC -3'	PrimerBank	PrimerBank ID: 6680868a1
Cdk4; forward 5'- ATGGCTGCCACTCGATATGAA -3', reverse 5'- TCCTCCATTAGGAACTCTCACAC -3'	PrimerBank	PrimerBank ID: 6753380a1
Cdk1; forward 5'- AGAAGGTACTTACGGTGTGGT -3', reverse 5'- GAGAGATTTCCCGAATTGCAGT -3'	PrimerBank	PrimerBank ID: 31542366a1
Ccnb1; forward 5'- AAGGTGCCTGTGTGTGAACC -3', reverse 5'- GTCAGCCCCATCATCTGCG -3'	PrimerBank	PrimerBank ID: 28195398a1
Cad; forward 5'- CTGCCCGGATTGATTGATGTC -3', reverse 5'- GGTATTAGGCATAGCACAAACCA -3'	PrimerBank	PrimerBank ID: 28175177a1
Smpdl3b; forward 5'- GCAGGGGCTCAACTAGGGA -3', reverse 5'- GGGGTCTTTGGATACGGTGTA -3'	PrimerBank	PrimerBank ID: 118130844c1
Cdc25a; forward 5'- TCCCTGACGAGAATAAATTCCCT -3', reverse 5'- TCGATGAGGTGAAAGGTGTCG -3'	PrimerBank	PrimerBank ID: 92373435c1
Polr3d; forward 5'- AAAAGCGTGAACGGGACAGG -3', reverse 5'- AATGGGACTGGATCACTTCCG -3'	PrimerBank	PrimerBank ID: 256225459c1
Bzw2; forward 5'- AGCGACTGTCTCAGGAATGC -3', reverse 5'- CTGTTTCCGGAAGGTCGTT -3'	This paper	N/A
Pinx1; forward 5'- AGCAAGGAGCCACAGAACATA -3', reverse 5'- GGTGAGCAATCCAGTTGTCTT -3'	This paper	N/A
7SK; forward 5'- GACATCTGTCACCCCATTGA -3', reverse 5'- GCGCAGCTACTCGTATACCC -3'	This paper	N/A
Polr3g; forward 5'- GCCCCTGAAAACGGGAGAAG -3', reverse 5'- TCGGGTGGTTCAATAAAGTACG -3'	PrimerBank	PrimerBank ID: 124487176c1
Gnl3; forward 5'- AAAGCGAGTAAACGTATGACCTG -3', reverse 5'- AGCACTATTTGGAACACCTGG -3'	PrimerBank	PrimerBank ID: 158517943c1
Mxd4; forward 5'- GGCGACTTCGCAAGGAAGAA -3', reverse 5'- GAGCCTGAGTTTAGCTCGTCT -3'	PrimerBank	PrimerBank ID: 31560565c1
Mxd3; forward 5'- GAGGCAGAGCACGGTTATG -3', reverse 5'- TGTAGTGTATCGGGTACAGTCAA -3'	PrimerBank	PrimerBank ID: 7710054a1
Mxi1; forward 5'- CACAATGAGTTGGAAAAGAACCG -3', reverse 5'- CCAGCGGGATCAGAACTTTCA -3'	PrimerBank	PrimerBank ID: 114520598c1
Mxd1; forward 5'- AGATGCCTTCAAACGGAGGAA -3', reverse 5'- CAAGCTCAGAGTGGTGTGTCG -3'	PrimerBank	PrimerBank ID: 6754604a1
Myh6; forward 5'- GCCCAGTACCTCCGAAAGTC -3', reverse 5'- GCCTTAACATACTCCTCCTTGTC -3'	PrimerBank	PrimerBank ID: 6754774a1
St6galnac4; forward 5'- TGGTCTACGGGGATGGTCA -3', reverse 5'- CTGCTCATGCAAACGGTACAT -3'	This paper	N/A
MycER; forward 5'- ATTTCTGAAGACTTGTTGCGGAAA -3', reverse 5'- GCTGTTCTTAGAGCGTTTGATCATGA -3'	Murphy et al., 2008	N/A
Tomato; forward 5'- ACCTCCCACAACGAGGACTA -3', reverse 5'- GAGGTGATGTCCAGCTTGGT -3'	This paper	N/A
Uck2; forward 5'- CGAAGGGATCCTAGCCTTCT -3', reverse 5'- TTTTCGCTGATGTCCCTGAAT -3'	This paper	N/A
Actin; forward 5'- GACGATATCGCTGCGCTGGT-3', reverse 5'- CCACGATGGAGGGGAATA-3'	This paper	N/A
Gapdh; forward 5'- AGGTCGGTGTGAACGGATTTG-3', reverse 5'- TGTAGACCATGTAGTTGAGGTCA-3'	This paper	N/A
