# Supplementary material for: Reactivation of Myc transcription in the mouse heart unlocks its proliferative capacity
Source: Nat Commun. 2020 Apr 14;11:1827. doi: 10.1038/s41467-020-15552-x (PMC7156407; doi:10.1038/s41467-020-15552-x)
Supplement: Supplementary file 3 — Reporting Summary [file 41467_2020_15552_MOESM3_ESM.pdf]

nature research

Corresponding author(s): Catherine Wilson & Gerard Evan

Last updated by author(s): 18/02/2020

Reporting Summary

Nature Research wishes to improve the reproducibility of the work that we publish. This form provides structure for consistency and transparency in reporting. For further information on Nature Research policies, see Authors & Referees and the Editorial Policy Checklist.

Statistics

For all statistical analyses, confirm that the following items are present in the figure legend, table legend, main text, or Methods section.

n/a	Confirmed	
☐	☒	The exact sample size (*n*) for each experimental group/condition, given as a discrete number and unit of measurement
☐	☒	A statement on whether measurements were taken from distinct samples or whether the same sample was measured repeatedly
☐	☒	The statistical test(s) used AND whether they are one- or two-sided<br>*Only common tests should be described solely by name; describe more complex techniques in the Methods section.*
☐	☒	A description of all covariates tested
☒	☐	A description of any assumptions or corrections, such as tests of normality and adjustment for multiple comparisons
☐	☒	A full description of the statistical parameters including central tendency (e.g. means) or other basic estimates (e.g. regression coefficient) AND variation (e.g. standard deviation) or associated estimates of uncertainty (e.g. confidence intervals)
☐	☒	For null hypothesis testing, the test statistic (e.g. *F*, *t*, *r*) with confidence intervals, effect sizes, degrees of freedom and *P* value noted<br>*Give P values as exact values whenever suitable.*
☒	☐	For Bayesian analysis, information on the choice of priors and Markov chain Monte Carlo settings
☒	☐	For hierarchical and complex designs, identification of the appropriate level for tests and full reporting of outcomes
☒	☐	Estimates of effect sizes (e.g. Cohen's *d*, Pearson's *r*), indicating how they were calculated

Our web collection on statistics for biologists contains articles on many of the points above.

Software and code

Policy information about availability of computer code

Data collection	No custom computer code or algorithm were used in this manuscript. qPCR data was collected in quantstudio design and analysis software v1.2. All IHC and IF images were captured in Zeiss AxioVision 4.8 software. TEM data was collected on Apreo FESEM equipped with a VolumeScope serial block face system and MAPS software (Thermo Fisher). qRT-PCR reactions were performed on a Applied Biosystems QuantStudio 5 Real-Time PCR System. Flow cytometry of MEFs was performed on a BD Accuri C6 flow cytometer. Flow cytometry of cardiomyocytes was performed on a BD LSRFortessa.
Data analysis	No custom computer code or algorithm were used in the data analysis of this manuscript. Flow cytometry analysis was performed in FlowJo software (Tree Star). RNAseq sample processing and analysis used the following software; TopHat aligner (version 2.0.8), Bioconductor package DESeq2, Cutadapt (version 1.11), BWA aln for short reads (version 2.5.2a), RSEM (version 1.2.30), edgeR package, Trimmomatic, STAR, HTSeq-count, DAVID and GENE-E (Broad Institute). Intron analysis was performed with the INSPEcT tool. All graphs were drawn in GraphPad Prism v6.0d. ChIPseq data analysis used the following softwear; BWA aligner, MACS software (v2.0.9) and Genomation R package. Bioinformatic and statistical analysis were performed using R with Bioconductor packages and comEpiTools packages. Motif analysis was performed using CentriMo software. Venn diagrams were draw in the interactive tool Venny. Gene lists from RNA and ChIP sequencing were analysed in Enrichr. TEM data was analysed in Imod (Bio3D) and Chimera (UCSF) software. Statistical analyses for IHC, heart/tibia ration, cardiomyocyte number and q-RTPCR were performed using GraphPad Prism.

For manuscripts utilizing custom algorithms or software that are central to the research but not yet described in published literature, software must be made available to editors/reviewers. We strongly encourage code deposition in a community repository (e.g. GitHub). See the Nature Research guidelines for submitting code & software for further information.

Data

Policy information about availability of data

All manuscripts must include a data availability statement. This statement should provide the following information, where applicable:

- Accession codes, unique identifiers, or web links for publicly available datasets
- A list of figures that have associated raw data
- A description of any restrictions on data availability

All datasets generated in this study, including Myc ChIP sequencing, RNA PolII ChIP sequencing and RNA sequencing, have been deposited in ArrayExpress (www.ebi.ac.uk/arrayexpress) under accession codes; E-MTAB-7592 [https://www.ebi.ac.uk/arrayexpress/experiments/E-MTAB-7592], E-MTAB-7593 [https://www.ebi.ac.uk/arrayexpress/experiments/E-MTAB-7593], E-MTAB-7595 [https://www.ebi.ac.uk/arrayexpress/experiments/E-MTAB-7595], E-MTAB-8462 [https://www.ebi.ac.uk/arrayexpress/experiments/E-MTAB-8462], E-MTAB-8515 [https://www.ebi.ac.uk/arrayexpress/experiments/E-MTAB-8515] and E-MTAB-7636 [https://www.ebi.ac.uk/arrayexpress/experiments/E-MTAB-7636]. The source data underlying Figs 1a,c d, 3a, 4a-e, 5a, b, d, g, h 6a-c, f, g and i, and, Supplementary Figs 1a-d, f, h 2a, 3a, b, 4a-f, 5b, d, h, 6 a, c f and g are provided as a Source Data file. Further information and requests for resources and reagents should be directed to, and will be fulfilled by, Gerard Evan (gie20@cam.ac.uk). Requests regarding AZ5576 should be directed to AstraZeneca PLC.

Field-specific reporting

Please select the one below that is the best fit for your research. If you are not sure, read the appropriate sections before making your selection.

☒ Life sciences ☐ Behavioural & social sciences ☐ Ecological, evolutionary & environmental sciences

For a reference copy of the document with all sections, see nature.com/documents/nr-reporting-summary-flat.pdf

Life sciences study design

All studies must disclose on these points even when the disclosure is negative.

Sample size	In accordance with Home Office guidelines experiments with mice were conducted with a minimum number of animals. Sample sizes were chosen based on extensive prior experience of the lab in characterising over expression model of Myc. A minimum of three biological replicates were used per experimental condition, except ChIP sequencing where two replicates were used and TEM where one sample was used.
Data exclusions	On very rare occasions where abnormally high Ct was observed in samples or a technical replicate, clearly resulting from RNA quality or pipetting error qPCR data was excluded.
Replication	The results of all in vivo experiments were reproducible as shown across multiple animals (exact n values indicated in the text and figures) over multiple cohorts. Where possible quantitative PCR, immunohistochemical, immunofluorescence and RNA sequencing analysis used at least three independent biological replicates with minimal variation. For ChIP sequencing analysis two replicates were used.
Randomization	Mice were bred in-house with groups being populated with mice as soon as they became available. Mice were randomly assigned to groups where applicable. Experimental groups provide a roughly equal mix of males and females.
Blinding	All immunohistochemical quantification were conducted with the investigator blind to sample identity.

Reporting for specific materials, systems and methods

We require information from authors about some types of materials, experimental systems and methods used in many studies. Here, indicate whether each material, system or method listed is relevant to your study. If you are not sure if a list item applies to your research, read the appropriate section before selecting a response.

Materials & experimental systems

n/a	Involved in the study	
☐	☒	Antibodies
☒	☐	Eukaryotic cell lines
☒	☐	Palaeontology
☐	☒	Animals and other organisms
☒	☐	Human research participants
☒	☐	Clinical data

Methods

n/a	Involved in the study	
☐	☒	ChIP-seq
☐	☒	Flow cytometry
☒	☐	MRI-based neuroimaging

Antibodies

Antibodies used

Rabbit monoclonal anti-GAPDH (D16H11) XP®, Cell Signalling Technology, Cat#: 5174
Mouse monoclonal anti-β-Actin (AC-15), Santa-Cruz Biotechnology, Cat#: sc-69879
Mouse monoclonal anti-α-Tubulin Antibody (DM1A), Santa-Cruz Biotechnology, Cat#: sc-32293
Rabbit monoclonal anti-Myc (Y69), Abcam, Cat#: ab32072
Mouse monoclonal anti-Rpb1 CTD (4H8), Cell Signalling Technology, Cat#: 2629
Rabbit monoclonal anti-Phospho-Rpb1 CTD (Ser2) (E1Z3G), Cell Signalling Technology, Cat#: 13499
Rabbit monoclonal anti-Phospho-Rpb1 CTD (Ser5) (D9N5I), Cell Signalling Technology, Cat#: 13523
Rabbit monoclonal anti-CDK9 (C12F7), Cell Signalling Technology, Cat#: 2316
Rabbit monoclonal anti-Cyclin T1 (D1B6G), Cell Signalling Technology, Cat#: 81464
Rabbit polyclonal anti-LARP7, Abcam, Cat#: ab134746
Rabbit monoclonal anti-Phospho-p44/42 MAPK (Erk1/2) (Thr202/Tyr204), Cell Signalling Technology, Cat#: 4370
Rabbit monoclonal anti-Ki67 (SP6), Thermo Scientific, Cat#: RM-9106-S1
Rabbit Polyclonal anti-phospho-Histone H3 (Ser10), Merck Millipore, Cat#: 06-570
Rabbit monoclonal anti-Cardiac Troponin T (13-11), Thermo Scientific, Cat#: MA5-12969
Rat monoclonal anti-BrdU [BU1/75 (ICR1)] antibody, Abcam, Cat# ab6326
Rabbit Polyclonal anti-Myc N262, Santa Cruz, Cat# sc-764X
Rabbit Polyclonal anti-RNA Pol II N20, Santa Cruz Cat# sc-899X
Rabbit Polyclonal anti-Aurora B antibody, Abcam, Cat# ab2254
Rabbit Polyclonal anti-PCM1, SigmaAldrich, Cat# HPA023370
Rat Monoclonal anti-Ki67 SolA15, Thermofisher Cat#14-5698-82

Validation

All commercial, common antibodies used consistent with the literature and data sheets.

Animals and other organisms

Policy information about studies involving animals; ARRIVE guidelines recommended for reporting animal research

Laboratory animals

The following mouse strains were used; R26LSL-CAG-c-MycERT2, R26LSL-c-MycER, Tg(Zp3-cre)93Knw, Gt(ROSA)26Sortm4(ACTB-tdTomato,-EGFP)Luo, and Tg(Myh6-cre)2182Mds/J. Mice were a mixture of males and females, collected at adulthood, 8-10 weeks old or juvenile 15-17 days old. Mouse embryo fibroblasts were collected at 13.5 days post fertilization.

Wild animals

This study did not involve wild animals.

Field-collected samples

This study did not involve samples collected from the field.

Ethics oversight

All experimental procedures received ethical approval and were conducted in accordance with the Home Office UK guidelines, under project licences 70/7586, 80/2396 (G.I.E.) which were evaluated and approved by the Animal Welfare and Ethical Review Body at the University of Cambridge.

Note that full information on the approval of the study protocol must also be provided in the manuscript.

ChIP-seq

Data deposition

☒ Confirm that both raw and final processed data have been deposited in a public database such as GEO.

☒ Confirm that you have deposited or provided access to graph files (e.g. BED files) for the called peaks.

Data access links
May remain private before publication.

Raw data generated in this study, including Myc ChIP sequencing and RNA PolII ChIP sequencing have been deposited in ArrayExpress under accession codes;E-MTAB-7592 [https://www.ebi.ac.uk/arrayexpress/experiments/E-MTAB-7592] and E-MTAB-7593 [https://www.ebi.ac.uk/arrayexpress/experiments/E-MTAB-7593].

Files in database submission

heart_A_wt_Myc.fastq.gz
heart_B_CAG_Myc.fastq.gz
heart_C_wt_Myc.fastq.gz
heart_D_CAG_Myc.fastq.gz
liver_A_wt_Myc.fastq.gz
liver_B_CAG_Myc.fastq.gz
liver_C_wt_Myc.fastq.gz
liver_D_CAG_Myc.fastq.gz
heart_A_wt_peaks
heart_B_CAG_peaks
heart_C_wt_peaks
heart_D_CAG_peaks
liver_A_wt_peaks
liver_B_CAG_peaks
liver_C_wt_peaks
liver_D_CAG_peaks
heart_75_3B.fastq.gz

heart_78_3C.fastq.gz
liver_75_3B.fastq.gz
liver_78_3C.fastq.gz

Genome browser session (e.g. UCSC)	no longer applicable

Methodology

Replicates	For ChIP sequencing two replicates were used.
Sequencing depth	Single end sequencing was performed on the Hiseq 2000. Reads were 50 bp. Total number of reads ranged from 31,491,714 to 59,728,939. After alignment on Mouse Genome (Assembly mm9) samples had between 13,944,268 to 54,379,305 reads per sample.
Antibodies	Rabbit Polyclonal anti-Myc N262, Santa Cruz, Cat# sc-764X<br>Rabbit Polyclonal anti-RNA Pol II N20, Santa Cruz Cat# sc-899X
Peak calling parameters	Data were generated and processed by aligning sequence reads to the mm9 genome with the BWA aligner using default settings. Peaks were called using the MACS software (v2.0.9). Normalized read counts within a genomic region were determined as the number of reads per million of library reads (total number of aligned reads in the sequencing library). Peak enrichment was determined as log2 (Peakw/Nc — inputw/Ni), where Peakw is the read count on the enriched region and inputw the read count on the same region in the corresponding input sample, Nc is the total number of aligned reads in the ChIP sample, and Ni is the total number of aligned reads in the input sample.
Data quality	ChIP seq reads were aligned using BWA aligner (HTS-flow default parameters). Peaks were called with MACS2 software (narrow and broad peaks, HTS-flow default parameters). Myc Peaks called for wild type heart and liver samples had an mean of 186 and 556 peaks respectively, Myc over expressing heart and liver samples had an mean of 29090 and 33004 peaks respectively.
Software	No custom computer code or algorithm were used in the data analysis of this manuscript. ChIPseq data analysis used the following softwear; BWA aligner, MACS software (v2.0.9) and Genomation R package. Bioinformatic and statistical analysis were performed using R with Bioconductor packages and comEpiTools packages.

Flow Cytometry

Plots

Confirm that:

- [ ] The axis labels state the marker and fluorochrome used (e.g. CD4-FITC).
- [ ] The axis scales are clearly visible. Include numbers along axes only for bottom left plot of group (a 'group' is an analysis of identical markers).
- [ ] All plots are contour plots with outliers or pseudocolor plots.
- [x] A numerical value for number of cells or percentage (with statistics) is provided.

Methodology

Sample preparation	For primary, non-immortalized murine embryonic fibroblasts were trypsinised, washed in PBS and fixed in 70% ethanol at 4 degrees overnight. Fixed cells were re-hydrated and washed in PBS prior to staining with propidium iodide in the presence of RNaseA.<br><br>For dissociated cariomyocytes, 500,000 cardiomyocytes were incubated with blocking buffer (4% BSA, +0.2% Triton X-100, +1mM EDTA, +0.02% sodium azide) and agitated (1000 rpm) at room temperature for 3 minutes. Cells were then re-suspend in primary antibodies (Cardiac Troponin T (13-11) (ThermoFisher, MA5-12969; 1:100, p-H3 (Millipore, 1:400) and incubated with agitation for 1 hour. After 2 washes in blocking buffer, secondary antibodies (Alexa Fluor 488 Goat Anti-mouse IgG (H+L) (Life Technologies, A11008)) were incubated together with Hoechst and agitated for 1 hour at room temperature. Following 2 washes in blocking buffer cells were re-suspend in blocking buffer and analysed by flow cytometry.
Instrument	BD Accuri C6 flow cytometer and BD LSRFortessa
Software	FlowJo (Tree Star)
Cell population abundance	n/a
Gating strategy	For MEFs the most dominant population in terms of morphology was identified on a FSC-A/SSC-A dot plot, the DNA content of this whole population was visualized on a histogram of FL3 (propidium iodide)-A. The frequency of DNA content reflecting the different cell cycle phases was determined using the Dean-Jett-Fox model. Within each sample the frequency of the G0/G1 phase + S-phase + G2/M phase was normalised to 100%.<br><br>For cardiomyocytes, events representing cells with viable DNA content were identified on a Hoechst/FSC-A dot plot.

Cardiomyocytes were identified by expression of cardiac troponin T (CTNT, Alexa-488) on a Alexa 488-A/FSC-A dot plot. The frequency of pH3+ve (Alexa-647) cardiomyocytes were identified on a Alexa 488-A/Alexa 647-A dot plot.

☒ Tick this box to confirm that a figure exemplifying the gating strategy is provided in the Supplementary Information.
