# Supplementary material for: Reactivation of Myc transcription in the mouse heart unlocks its proliferative capacity
Source: Nat Commun. 2020 Apr 14;11:1827. doi: 10.1038/s41467-020-15552-x (PMC7156407; doi:10.1038/s41467-020-15552-x)
Supplement: Supplementary file 4 — Description of Additional Supplementary Files [file 41467_2020_15552_MOESM4_ESM.pdf]

Description of Additional Supplementary Files

Supplementary Data 1.

Differentially expressed genes in the kidney, liver, lung (n=3) and heart (n=5) of *R26*$^{CMER/+}$ mice in comparison to wild-type (*R26*$^{+/+}$), as determined by RNA sequencing (FDR<0.05 and abs(log2FC)>0.5) at 4 hours post administration of 4-OHT.

Supplementary Data 2.

Enrichr RNA sequencing analysis.

Top 6 Mouse Gene Atlas Gene sets and MGI Mammalian Phenotype Gene sets that significantly overlap with the list of genes that show a differential decrease in expression (DOWN DEGs) only in the kidney (Kidney specific, yellow, n=3) or lung (Lung specific, blue, n=3) or heart (Heart specific, red, n=5) of *R26*$^{CMER/+}$ mice in comparison to wild-type (*R26*$^{+/+}$), as determined by RNA sequencing at 4 hours post administration of 4-OHT.

Supplementary Data 3.

Differentially expressed genes in the heart of 15 day *R26*$^{CMER/+}$ (n=3) mice in comparison to wild-type (*R26*$^{+/+}$, n=3), as determined by RNA sequencing (FDR<0.05 and abs (log2FC)>0.5) at 4 hours post administration of 4-OHT.

Supplementary Data 4.

Differentially expressed genes in the heart of adult mice, 4 weeks post AAV-CCNT1 infection, *Myh6-cre; R26*$^{LSL\text{-}CMER/+}$ (n=3) mice in comparison to; *R26*$^{LSL\text{-}CMER/+}$ (n=3), as determined by RNA sequencing (adjp <0.05 and abs (log2FC)>0.5) at 4 hours post administration of 4-OHT.

Supplementary Data 5.

Differentially expressed genes in purified cardiomyocytes of adult mice, 4 weeks post AAV-CCNT1 infection, Myh6-cre; *R26*$^{LSL\text{-}CMER/+}$ (n=3) mice in comparison to; *R26*$^{LSL\text{-}CMER/+}$ (n=3), as determined by RNA sequencing (adjp <0.05 and abs (log2FC)>0.5) at 4 hours post administration of 4-OHT.

Supplementary Data 6.

Differentially expressed genes from heart of adult mice, 4 weeks post AAV infection in R26-CMER/+; AAV-RFP (n=3) mice in comparison to; R26-CMER/+; AAV-CCNT1 (n=3), as determined by RNA sequencing (adjp <0.05 and abs (log2FC)>0.5) at 44 hours post administration of tamoxifen.

Supplementary Movie 1.

Movie file generated from 500 aligned TEM images produced using the 3View system of *Myh6-Cre*;*R26*$^{LSL\text{-}CMER/+}$ heart tissue from a single mouse 48 hours post administration of tamoxifen.

Supplementary Movie2.

Movie file of 3D visualization of cardiomyocytes in cytokinesis from *Myh6-Cre*;*R26*$^{LSL\text{-}CMER/+}$ heart tissue 48 hours post administration of tamoxifen. Using IMOD 3D reconstruction and visualization software models were assembled from Z-stack images. Green-outline of cells, blue-nuclei. Pick shows site of cross section through cells shown in Figure 5i.
